# *Aeromonas* Isolates from Human Diarrheic Stool and Groundwater Compared by Pulsed-Field Gel Electrophoresis

**DOI:** 10.3201/eid0902.020031

**Published:** 2003-02

**Authors:** Mark A. Borchardt, Mary E. Stemper, Jon H. Standridge

**Affiliations:** * Marshfield Medical Research Foundation, Marshfield, Wisconsin, USA; † Marshfield Laboratories, Marshfield, Wisconsin, USA; ‡ University of Wisconsin, State Laboratory of Hygiene, Madison, Wisconsin, USA

**Keywords:** diarrhea, drinking, water microbiology, electrophoresis, gel, pulsed-field, *Aeromonas*, bacterial typing techniques, research

## Abstract

Gastrointestinal infections of *Aeromonas* species are generally considered waterborne; for this reason, *Aeromonas hydrophila* has been placed on the United States Environmental Protection Agency Contaminant Candidate List of emerging pathogens in drinking water. In this study, we compared pulsed-field gel electrophoresis patterns of *Aeromonas* isolates from stool specimens of patients with diarrhea with *Aeromonas* isolates from patients’ drinking water. Among 2,565 diarrheic stool specimens submitted to a Wisconsin clinical reference laboratory, 17 (0.66%) tested positive for *Aeromonas*. Groundwater isolates of *Aeromonas* were obtained from private wells throughout Wisconsin and the drinking water of *Aeromonas*-positive patients. The analysis showed that the stool and drinking water isolates were genetically unrelated, suggesting that in this population *Aeromonas* gastrointestinal infections were not linked with groundwater exposures.

The Safe Drinking Water Act amendment of 1996 requires the United States Environmental Protection Agency (EPA) to establish a list of contaminants of public health concern that are known or anticipated to occur in drinking water systems and may require future regulation under the Safe Drinking Water Act. The list, known as the Contaminant Candidate List, is intended to generate scientific research that will assist the EPA in creating new regulations to protect the public from health risks associated with drinking water. Currently, the putatively emerging enteric pathogen, *Aeromonas hydrophila*, is included in the list because it has the potential to grow in water distribution systems, especially in biofilms, where it may be resistant to chlorination (*1*). However, the role of drinking water consumption in *Aeromonas* infections is unclear.

Three phenotypically defined species, *A. hydrophila*, *A. caviae*, and *A. veronii biotype sobria*, constitute 85% of all clinical isolates involved with gastrointestinal and extraintestinal infections (*2*). Whether *Aeromonas* is indeed a causative agent of gastroenteritis has been debated. Numerous case reports have described isolating *Aeromonas* from patients with acute diarrhea, but the bacterium can also be isolated from stool of healthy persons (*3*). Determining the enteropathogenicity of *Aeromonas* has been inconclusive, probably because of differences in diarrheagenic potential among strains. A consensus appears to be growing that certain strains are likely human enteric pathogens (*2*,*4*).

*Aeromonas* is ubiquitous in water, including chlorinated drinking water (*5*–*7*). In surface water, *Aeromonas* abundance peaks in the warm summer and fall months (*8*,*9*). In one municipality, the seasonal increase in *Aeromonas* detection in the drinking water supply matched the peak occurrence of clinical isolates (*8*). *Aeromonas* also occurs in groundwater (*6*,*10*,*11*), and in a single well, the same strain can persist for years (*11*). Some strains of *Aeromonas* isolated from water have been shown to possess virulence traits, such as adhesions, hemolysins, and cytotonic enterotoxins, presumably involved with human pathogenicity (*3*,*12*,*13*).

If *Aeromonas* enteric infections are transmitted by drinking water and symptomatic infections are strain-specific, then the same strains isolated from patients with acute gastroenteritis should be found in drinking water. The objective of this study was to isolate *Aeromonas* from patients with acute diarrhea and, by using pulsed-field gel electrophoresis (PFGE), compare the molecular fingerprints of these isolates with isolates from the patients’ drinking water.

## Methods

### Fecal Specimens

This study was reviewed and approved by the Institutional Review Board of Marshfield Clinic. All diarrheic stool specimens submitted by physicians to Marshfield Laboratories, a clinical reference laboratory, for routine microbiologic analysis were screened for *Aeromonas* during two periods, July 28–November 13, 1998, and June 2–October 18, 1999. Specimens were plated for *Aeromonas* within 2–3 days after submission. Stool in Cary-Blair transport media was directly streaked to sheep blood agar containing 10 μg/mL ampicillin (*14*) (Remel, Lenexa, KS) and incubated at 35°C. Presumptive *Aeromonas* isolates were screened for standard phenotypic traits (β-hemolysis, oxidase positive, indole positive), and species identity was determined by using the API-20E identification system (10th edition, analytical profile index, bioMérieux, Vitek, Marcy-‘Etoile, France).

### Drinking Water Samples

Patients with positive results for *Aeromonas* were asked to allow a trained technician to collect a water sample from their residence. Samples were collected within 1–3 weeks after the clinical isolate was identified. *Aeromonas* was directly cultured from two 100-mL water samples by using ampicillin dextrin agar in a membrane filtration technique (*15*). One sample was incubated at 30°C and the other at 35°C. Yellow oxidase-positive colonies were streaked for purity and confirmed as *Aeromonas* by using the API-20E (bioMérieux) identification system. Stool and water isolates were stored in Microbank cryovials (Pro-Lab Diagnostics, Richmond Hill, Ontario, Canada) at –70°C for subsequent PFGE.

### PFGE

The PFGE procedure for *Aeromonas* was modified from methods previously described (*16*,*17*). Isolates were grown overnight in 5 mL of brain heart infusion broth at 37°C, harvested by centrifugation, and washed with 1 mL resuspension buffer (10 mM Tris-HCl [pH 7.6], 1 M NaCl). Pelleted cells were adjusted to a concentration of 1 x 10^9^ CFU/mL in resuspension buffer by using a Vitek colorimeter (Hach Co., Loveland, CO), mixed with an equal volume of 2% low melt agarose (FMC BioProducts, Rockland, ME), dispensed into plug molds (Bio-Rad Laboratories, Hercules, CA), and allowed to solidify 10 min at room temperature. Plugs were incubated in 3 mL lysis buffer (6 mM Tris-Cl, 1.0 M NaCl, 0.1 M EDTA, 0.5% Brij 58, 0.5% sarkosyl, 0.2% deoxycholate, 1 mg/mL lysozyme) at 37°C for 4 h. Lysis buffer was replaced with proteinase K solution (0.5 M EDTA, 1% N-lauroyl sarcosine, 1mg/mL proteinase K) followed by incubation at 55°C overnight. Plugs were washed 3 times in Tris-EDTA buffer (10 mM Tris-HCl, 0.1 M EDTA [pH 8.0]) and stored at 4°C. Genomic DNA was digested with 30 U *Xba*I (Promega Corp., Madison, WI) at 37°C overnight. Electrophoresis was performed in 1% Seakem agarose (FMC Bioproducts) by using the CHEF-DRIII system (Bio-Rad Laboratories) in 0.5× TBE buffer (45 mM Tris, 45 mM boric acid, 1 mM EDTA [pH 8.0]) at 14°C. The running parameters were 150 V for 12 h with 20-sec pulses and 17 h with 5- to 15-sec pulse times. One *A.* *hydrophila* isolate (isolate 1,320) was run in multiple lanes of each gel as a DNA global reference for standardizing runs. DNA band size was determined from *Staphylococcus aureus* strain NCTC 8325 DNA, digested with *Sma*I. DNA banding patterns were visualized with 0.1% ethidium bromide and digitally photographed. Molecular Analyst Fingerprinting Plus software (version 1.12, Bio-Rad Laboratories) was used to compare the genetic similarity among isolates and construct a similarity dendrogram by using the Dice coefficient and the UPGMA algorithm (unweighted pair-group method with arithmetic mean) with a position tolerance of 1.5%.

## Results

**Figure 1:**
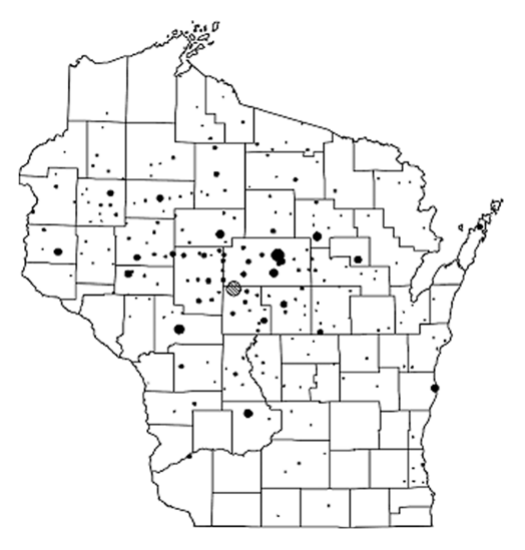
Location of Wisconsin residents who submitted diarrheic stool specimens to Marshfield Laboratories. The symbol 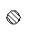 indicates the location of Marshfield, WI. Symbol size is proportional to the number of specimens. (For reference, the symbol for Marshfield = 208 specimens.)

Cultures for *Aeromonas* were performed on 2,565 diarrheic stool specimens from 2,310 patients. The median age of the patient population was 37 years (range 4 days to 97 years), and 55% were female. Most specimens (97.6%) were from patients residing in Wisconsin, primarily the central portion of the state, where groundwater, either from a municipal system or private well, is the source of drinking water (Figure 1). Some specimens came from communities along Lake Michigan where lake water is the source of drinking water.

Seventeen specimens (0.66%) from 17 patients (0.74%) tested positive for *Aeromonas*. Three stool isolates were identified as *A. hydrophila* and 14 isolates were *A. caviae*. All positive specimens were from Wisconsin residents. The median age of *Aeromonas*-positive patients was 27 years (range 1 month to 87 years) and 59% were male. Five *Aeromonas*-positive patients were coinfected with one other enteric pathogen (two patients with *Campylobacter*, one with *Salmonella,* one with *Cryptosporidium,* and one with *Clostridium difficile* toxin A), suggesting that in these patients *Aeromonas* may have been a transient colonizer.

Fourteen of the *Aeromonas*-positive patients agreed to have their drinking water sampled. Five patients resided in a household with a private well, eight were served by municipal wells, and one lived in a municipality that used Lake Michigan for its drinking water. Except for one system, all municipal water was chlorinated. One drinking water source, a private well, tested positive for *A. hydrophila*. Designing this study, we assumed that the fecal carriage rate of *Aeromonas* would be similar to its carriage rate in another study conducted in the Midwest (*18*) and, based on the ubiquitous occurrence of *Aeromonas* in water, we anticipated that a number of isolates would be collected from patients’ drinking water. Since only one was collected, additional drinking water isolates were obtained by combining samples from 1,500 private wells throughout Wisconsin that had been submitted to the Wisconsin State Laboratory of Hygiene between September and November 1998 for routine coliform testing. The composite samples (composed of 4–10 well samples) were membrane filtered and cultured for *Aeromonas* as described above. This process yielded an additional 37 *A.* *hydrophila* and 17 *A. caviae* isolates.

**Figure 2:**
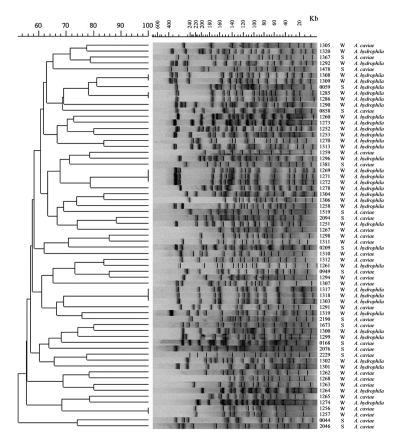
Pulsed-field gel electrophoresis patterns and similarity dendrogram of genomic DNA from *Aeromonas* *hydrophila* and *A. caviae* isolates from diarrheic stool (S) and groundwater (W). The number refers to the isolate number. DNA molecular weight scale derived from *Staphylococcus aureus* NCTC 8325.

PFGE of the stool and groundwater *Aeromonas* isolates yielded 10–20 well-resolved genomic DNA bands, ranging in size from approximately 10–400 kb (Figure 2). Six isolates (one stool, five groundwater) were not amenable to *Xba*I digestion, resulting in poorly resolved DNA fragments. PFGE patterns indicated extensive genetic diversity. The 65 isolates analyzed by PFGE had 58 distinct patterns. Five patterns grouped two or more identical isolates. Three of those groups included only isolates derived from the same composite water sample, suggesting multiple isolations of the same strain. Analyzing all pairwise comparisons among the 65 isolates, the median similarity was 59% (range 16% to 100%, n=2,080). Isolates from the same ecologic source also exhibited high genetic diversity. The median similarities of stool and groundwater isolates were 58% (range 30% to 76%, n=120) and 60% (range 25% to 100%, n=1,176), respectively. Among the 12 isolates from diarrheic stool specimens that were negative for other enteric pathogens, the median similarity was 58% (range 30%–76%, n=66).

None of the stool isolates was genetically indistinguishable from the groundwater isolates (Figure 2). The two isolates that appeared epidemiologically related, the patient stool isolate (isolate 0209) and the isolate from his private well (isolate 1320), were 72% similar by the Dice coefficient and differed by nine bands, which, following the criteria of Tenover et al. (*19*), would be generally interpreted as being genetically unrelated. The highest similarity between a stool and water pair of isolates was 88%, between isolates 1251 and 2094, which differed by four bands (Figure 2). However, the stool isolate was from a patient simultaneously positive for *Cryptosporidium* oocysts, suggesting that the *Aeromonas* was transient. The second highest similarity was 86%, a four-band difference between isolates 0949 and 1294 (Figure 2). Of the 784 paired comparisons between stool and water isolates, 776 (99%) had similarities <80%, and the median similarity was 58% (range 16% to 88%).

## Discussion

The prevalence rate in this study was lower than rates from other large surveillance studies in the United States for *Aeromonas* in stool. *Aeromonas* was cultured from 2,848 diarrheic stool specimens submitted to a Los Angeles, California, hospital in the early 1980s and 80 (2.8%) were positive (*20*). Among 1,821 patients with diarrhea visiting a clinic in La Crosse, Wisconsin, during an 18-month period, Agger et al. (*21*) identified 20 (1.1%) that were positive for *A.* *hydrophila*. Moyer (*18*) examined 3,334 diarrheic stool specimens submitted by physicians over a 2-year period to an Iowa public health laboratory and found 238 (7.1%) positive for either *A. caviae, A. hydrophila*, or *A. sobria*. Isolation in the latter study included an alkaline peptone water enrichment step, which may explain the higher prevalence rate. In our study, the specimen prevalence rate was 0.66%, and this rate was likely biased upwards because stool specimens were collected only in the summer and fall months when the incidence of *Aeromonas* gastrointestinal infections is reportedly highest (*18*,*21*). The data, albeit limited, do not suggest that the prevalence of *Aeromonas* enteric infections is increasing in the United States. Worldwide, the isolation rate of *Aeromonas* from diarrheic stool has been reported as high as 10.8% (*22*) and as low as 0% (*23*). In the latter study, recently conducted in Melbourne, Australia, during a 68-week observation period, 795 fecal specimens were collected from city residents with highly credible gastroenteritis. *Aeromonas* was not detected in any of the fecal specimens, even though 50% of water samples drawn weekly from the drinking water distribution mains serving the study participants were *Aeromonas*-positive (*23*).

The *A. hydrophila* and *A. caviae* species designations were equivocal in this study. Except for stool isolate 0858, all isolates from stool identified by the API-20E system as *A. caviae* were β-hemolytic, suggesting that they were *A. hydrophila* instead. When the Vitek automated microbe identification system (bioMérieux) was used, all stool and water isolates were identified as the *A.* *hydrophila*/*caviae* group. If the most recent edition of the API analytical profile index were used with the *A. caviae* profiles derived during the study, the new species designation would be *A. hydrophila* group 1. Some profiles determined in this study are not listed in the most recent index and, given the ever-changing taxonomy of aeromonads (*4*), we opted for a consistent one-index approach, reporting the species designations for all stool and water isolates on the basis of the index available at the time of the study.

The high level of genetic diversity observed in our study among clinical and environmental strains of *Aeromonas* has been corroborated by other nucleic acid–based subtyping methods, such as amplified fragment length polymorphisms (*11*) and ribotyping (*24*). Talon et al. (*17*) subtyped 10 epidemiologically unrelated strains of *A.* *hydrophila* by PFGE and reported that the median similarity (calculated by using the Pearson correlation coefficient) was 28.4% (range 9.3% to 44.3%). The variation is not likely due to genetic lability, because the PFGE patterns of *Aeromonas* reportedly do not become unstable during frozen storage and long-term laboratory culture (*17*,*25*). As a control in this study, isolate 1320 was digested and underwent electrophoresis five independent times, and each time yielded the same PFGE pattern. The PFGE patterns also did not correspond to phenospecies. Millership and Want (*26*) reported a similar finding based on whole-cell protein fingerprinting.

The capacity for human enteropathogenicity among clinical isolates may be derived from a unique set of genes that were acquired or evolved in a common ancestor. Alternatively, enteropathogenicity may have arisen independently among several genotypes. In either scenario, one might expect the subset of *Aeromonas* strains that are pathogenic to have less genetic variation than the environmental strains. In this study, the level of genetic diversity was similar between environmental and clinical strains, even when the clinical strains were restricted to the subset from stool specimens that were negative for other enteric pathogens. One explanation is that some clinical strains were transient gastrointestinal colonizers, and only a few strains that are truly diarrheagenic. This issue will likely remain unresolved until the pathogenicity mechanisms of *Aeromonas* are better understood.

PFGE is a reproducible, highly discriminatory subtyping method capable of identifying the transmission source of bacterial infections (*19*). If the *Aeromonas*-positive patient with the positive well acquired the infection from his drinking water, the molecular fingerprints of the stool and water isolates should at least have been closely related (i.e., <3-band difference). Likewise, if drinking water is a frequent source of *Aeromonas* infections in this study population, one would expect at least a few of the stool and water isolates to be more closely related than a four-band difference. The analysis for this study was weighted towards identifying similar stool and water isolates. The 1.5% position tolerance that was selected for band calling resulted in matching bands that differed by as much as 17 kb, and the Dice coefficient gives greater weight to matching bands compared to other similarity coefficients.

The study had several limitations, however, that need to be considered when interpreting the data. Only one isolate per stool specimen or water sample was analyzed by PFGE. Multiple strains may have been present in water, and by chance the enteropathogenic strains were not selected for PFGE analysis. Kühn et al. found multiple *Aeromonas* strains in a single drinking water source, although only one or a few strains were numerically dominant, and these could persist for years (*6*,*11*). When duplicate water samples (incubated at 30°C and 35°C) both yielded *Aeromonas*, then PFGE was performed on a colony from each plate. Some of these isolate pairs were indistinguishable by PFGE, and some were unrelated, showing that some composite water samples had at least two different strains. Both strains were included in the genetic similarity analysis. Another study limitation was that if diarrheagenic *Aeromonas* strains are very rare in groundwater, the sample size might have been insufficient to find those strains, even though the composite water samples tested represented 1,500 wells. The stool and water isolates were collected from the same geographic area and during the same period, which should have increased the odds of finding genetically similar isolates if the two ecologic sources are linked. Finally, the drinking water sample volume for *Aeromonas* isolation was 100 mL. Since the study was conducted, the EPA has developed and validated Method 1605 for detecting *Aeromonas* in drinking water, which specifies a minimum sample volume of 1 L (*27*). Possibly, if the sample volume had been 1 L, more patient wells would have been *Aeromonas* positive.

To our knowledge, this is the first study to use PFGE to compare *Aeromonas* strains from human stool with strains found in groundwater. Other studies have compared the relatedness of strains from drinking water and stool by fatty acid methyl ester profiles (*28*), ribotyping (*29*), and randomly amplified polymorphic DNA (*30*), all highly discriminatory subtyping methods, and found little similarity between clinical and environmental isolates. *Aeromonas* isolates from stool and drinking water have been linked by biotyping (*31*) and whole-cell protein fingerprinting (*25*), but the discriminatory power of these methods with *Aeromonas* is questionable (*28*,*30*). Thus, the evidence to date from using highly discriminatory subtyping methods suggests that human enteropathogenic strains are rare in drinking water. In the group of primarily Wisconsin residents in this study, *Aeromonas* was identified infrequently in diarrheic stool specimens and drinking water from a groundwater source did not appear to be an *Aeromonas* transmission route.
